# Study on the environmental adaptation characteristics of red panda release into the wild

**DOI:** 10.1371/journal.pone.0331776

**Published:** 2025-10-01

**Authors:** Yanshan Zhou, Chao Chen, Xiang Yu, Wenlei Bi, Rui Ma, Wei Wu, Jiabin Liu, Guanwei Lan, Haijun Gu, Qian Zhang, Kunlin Yang, Liqiang Fu, Hong Pan, Dunwu Qi

**Affiliations:** 1 The Conservation of Endangered Wildlife Key Laboratory of Sichuan Province, Chengdu Research Base of Giant Panda Breeding, Chengdu, Sichuan, China; 2 Sichuan Forestry and Grassland Bureau, Chengdu, Sichuan, China; 3 Mabian Dafengding National Nature Reserve Conservation Center, Leshan, Sichuan, China; 4 Mabian Yi Autonomous county forestry Bureau, Leshan, Sichuan, China; Feroze Gandhi Degree College, INDIA

## Abstract

Animals undergo a cognitive process when exposed to novel environments, which plays a crucial role in their ability to identify optimal habitats and support long-term survival. We conducted an initial investigation into the spatial utilization and habitat selection patterns of a femal red panda using GPS collar technology. Our research revealed that the home range and core activity area of the red panda was larger during initial 60 days after release, and markedly decreased thereafter. The red panda’s selection of altitude did not align with that of wild individuals until 60 days after release, whereas slope selection may require at least 30 days to stabilize and become consistent with wild individuals’ patterns. Our study further revealed that the home range and core activity area of the red panda showed low overlap with the suitable habitat of the wild population during the initial two months; however, this overlap increased significantly, reaching over 90% thereafter. We hypothesize that the red panda may require at least 60 days to acclimate to its new environment after release. Although our study was only based on a single individual, it provides the first evidence of the environmental adaptation process of red panda following released into the wild, thereby establishing a crucial foundation for future conservation and reintroduction initiatives.

## Introduction

The acclimation period is a critical indicator for assessing an animal’s ability to survive and reproduce after being introduced to a new environment [1]. The selection of habitat serves as a pivotal factor in determining the balance between animal behavior and survival costs [2,3], whereas space utilization reflects the complex interplay between wildlife activity patterns and environmental resource allocation, thereby exerting profound influences on gene flow, population viability, and ecological dynamics [4,5]. Investigating the spatial utilization and habitat selection of wildlife is of considerable biological significance, as it enables a more comprehensive analysis of their resource use patterns and deepens our understanding of the adaptive strategies they employ in response to environmental conditions [6]. For instance, a detailed examination of migration patterns and home range can clarify the interactions among individuals within a population, thereby deepening our understanding of the trade-offs between an animal’s requirements and its surrounding environment [7,8].

The red panda (*Ailurus styani*) is a distinctive endangered species endemic to the Hengduan Mountains, and animal ecologists have conducted extensive research to elucidate its ecological characteristics and adaptive strategies in response to environmental conditions [9–11]. The red panda inhabit high mountain valleys, and its heightened alertness complicates direct observation in its natural habitat. Consequently, most previous research data have been derived from indirect evidence such as faeces and footprints [7,12]. As a result, GPS collar tracking technology has facilitated a comprehensive understanding of their behavioral characteristics, enabling studies on home ranges, migration patterns, and activity rhythms, among others aspects [13]. Compared to the giant pandas inhabiting the same geographic range, studies on the behavioral ecology of red pandas remain relatively scarce, with a primary focus on habitat and niche selection characteristics within wild populations [14–16]. In recent decades, the persistent impact of human activities has led to significant degradation of global forest ecosystems, thereby placing the survival of red pandas under severe threat. There has been a marked increase in cases involving the rescue and rehabilitation of red pandas with the aim of eventual release [17]. Prior to this study, there were limited reports on the adaptability of red panda to its environment.

It is widely acknowledged that different species exhibit distinct ecological requirements, necessitating specific environments to support their fundamental survival [18]. In contrast to the restricted environments of captivity, the natural habitats of most wildlife exhibit considerable complexity, with individuals demonstrating a preference for specific habitat types within their activity ranges [19–20]. Animals undergo an acclimatization period upon entering a new environment, during which their cognitive awareness of the surroundings develops and their utilization of the habitat transitions from instability to stability [21]. For instance, the wild red pandas select habitats characterized by bamboo distribution, gentle slopes, higher elevations, and moderate vegetation coverage for their activities [7,15,22,23]. Similar to other species, red pandas exhibit adaptive behaviors such as predator avoidance, foraging, resting, and reproduction to enhance their survival strategies and minimize energetic costs during the process of environmental adaptation [24]. Ultimately, they improve their ability to thrive in new environments by maximizing opportunities for survival [7,11,25]. For red pandas released into the wild, selecting optimal habitats within complex ecosystems represents a critical survival skill that is equally important for the dynamics of local wild populations [26–28]. Therefore, to ensure the successful survival and integration of released red pandas into local wild populations, it is essential for them to adapt to their natural environment and develop habitat selection skills similar to those of their wild counterparts [29]. This study analyzed the spatial distribution and habitat selection patterns of a rescued red panda in the wild, and explored its environmental adaptation process after release. The objective was to provide a theoretical basis for future research on the behavioral ecology of red pandas. Although our study was based on a single individual and could not yield statistically representative conclusions, the preliminary findings still hold significant reference value for advancing ecological research and conservation practices for this species.

## Methods

### Study area

Our study was conducted in the Liangshan Mountain Range of Sichuan Province, China, specifically within the Mabian Dafengding National Nature Reserve and Meigu Dafengding National Nature Reserve (103°5′E-103°26′E, 28°26′N-28°45′N). The area is situated on the southwestern edge of the Sichuan Basin and serves as a transitional zone between the Sichuan Basin and the Yunnan-Guizhou Plateau. It represents one of the primary distribution regions for the Chinese red panda in China [30].

The area where we tracked the released red panda was located the junction of Mabian and Meigu Dafengding National Nature Reserve. It covered a total area of 150 km^2^, with an elevation range of approximately 1,800–3,400 m and slopes varying from 6°to over 60°. According to China’s national fourth giant panda survey, wild red pandas were present in this region [31]. Within our study area, four distinct forest types were identified: evergreen broadleaf forests, deciduous broadleaf forests, mixed conifer-broadleaf forests, and subalpine coniferous forests [30]. The understory vegetation included arrow bamboo (*Sinarundinaria fangiana*), Qiong bamboo (*Qiongzhurea tumidinoda*), and Yushan bamboo (*Fargesia yunnanensis*) [32]. Additionally, the area is inhabited by other sympatric mammals including giant panda (*Ailuropoda melanoleuca*), black bear (*Ursus thibetanus*), yellow-throated marten (*Martes flavigula*), and leopard cat (*Prionailurus bengalensis*); however, these species do not pose a threat to adult red panda [30].

### Study subject and GPS collar installation

In April 2022, we rescued a total of 28 red pandas. Based on relevant rescue and release regulations, after approximately one year of captive care and rehabilitation, they were released into Mabian Dafengding National Nature Reserve in Sichuan Province, China, in May 2023. The release site was located approximately 30 km south of the original rescue location. Given the strict management practices of the nature reserve and minimal human disturbance, we decided against conducting the release at the initial rescue site. Prior to their release, we fitted three healthy adult red pandas (two females and one male) with GPS collars to facilitate the post-release monitoring of their environmental adaptation post-release. The collar-fitting protocol was subject to a comprehensive review and received formal approval from the Chengdu Research Base of Giant Panda Breeding Institutional Animal Care and Use Committee (IACUC NO. 2023.0328.0003). Unfortunately, among the three individuals fitted with GPS collars, two (one female and one male) experienced premature collar detachment after one month of operation, resulting in incomplete data collection. In contrast, the collar of the remaining female red panda functioned continuously for 210 days until it automatically detached due to battery depletion. Therefore, this study focused on this particular female red panda (the released individual) to preliminarily investigate her adaptation period in the new wild environment.

The GPS collar used in this study was designed for the Iridium Track M series (LotekWirelessInc., Ontario, Canada). Weighing 0.1 kg, the collar accounted for only 1.35% of the body mass of the red panda, which weighed 7.4 kg. Previous research suggestsed that collars weighing less than 5% of an animal’s body mass had minimal impact on its behavior [33]. Data were collected every 4 hours and transmitted daily via the Iridium satellite. This dataset includesd date, time, latitude and longitude coordinates, altitude, PDOP (Position Dilution of Precision), and ambient temperature.

We acknowledge the importance of following the “ARRIVE Guide” to improve the quality and academic integrity of animal research and hereby declare that this study is in full compliance with its requirements.

### Data collection and preprocessing

We collected the location data of the released individual from the Lotek WebService and excluded entries with unreliable positions based on PDOP values, fix status, and missing information. A total of 415 valid 3D location records were collected and categorized into seven time intervals according to post-release monitoring periods (0–30 days, 31–60 days, 61–90 days, 91–120 days, 121–150 days, 151–180 days, and 181–210 days) for subsequent statistical analysis (S1 Table). Additionally, prior to the release of red pandas, we collected 61 location records from wild red pandas within the release area through field surveys (S2 Table). These data consisted solely of latitude and longitude coordinates derived from foraging and faeces traces.

### Analysis of home range

We used the minimum convex polygon (MCP) and kernel density estimation (KDE) methods to evaluate the home range and core activity area (also referred to as the core home range) of the released individual across different post-release periods, implementing the analysis through the “Home Range Tools” extension in ArcGIS 10.8. Specifically, the home range was defined as the area encompassing 95% probability density contour lines of the individual’s utilization distribution, while the core activity area was defined as the region representing 50% probability density contour lines of the utilization distribution [34,35].

### Topographic variables

The topographic factors, including altitude, slope, and aspect, were derived from a digital elevation model (DEM) with a spatial resolution of 30 m × 30 m obtained from the Geospatial Data Cloud (http://www.gscloud.cn/) [35,36]. Slope and aspect data were extracted from the DEM layer using the Spatial Analyst in ArcGIS 10.8 [35] (S1 and S2 Tables). The Kruskal-Wallis test was applied to evaluate differences in terrain factors across various post-release periods, and a comparison was conducted between the released individual and its wild counterparts.

### Evaluation of habitat suitability

We used the MaxEnt model to evaluate habitat suitability for wild individuals within the study area. Based on field survey data on the location records of wild individuals, combined with plant information, remote sensing imagery, and other relevant data, we selected vegetation characteristics, topographic factors, and human disturbance as environmental variables for modeling [37]. We used the Normalized Difference Vegetation Index (NDVI) with a spatial resolution of 500 meters, obtained from the Geospatial Data Cloud (http://www.gscloud.cn), as a proxy for the vegetation characteristics variable in our modeling [38]. The MODND1 M data covering the period from May to December 2023 were selected, and the NDVI data were extracted using ENVI 5.5 software [39]. In ArcGIS 10.8, we performed Euclidean distance calculations to determine the distances from each grid cell within the study area to the nearest road and settlement. These distances were used as human disturbance variables in the modeling process [40].

## Results

### Temporal variations in home range and core activity area after release

**Fig 1:**
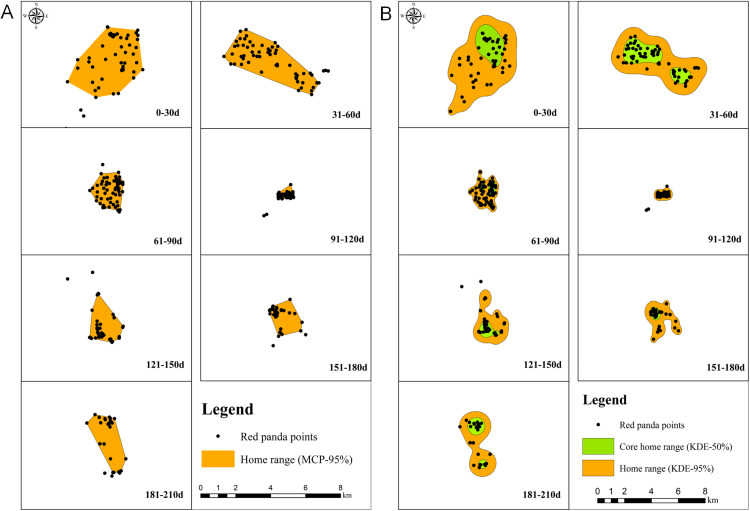
The estimation of the home range and core activity area of a female red panda released into the wild. A: The home range of the red panda, estimated using the MCP method after release. B: The home range and core activity area of the red panda, estimated using the KDE method after release.

We used MCP method to estimate the home range of the red panda across various time intervals after release (Fig 1A). The results indicated that the red panda exhibited a larger home range during the first 60 days post-release, with an area of 11.40 km^2^ in the 0–30 days and 9.13 km^2^ in the 31–60 days. However, there was a significant reduction in the home range to below 4 km^2^ after 60 days. The activity ranges were recorded as follows: 2.96 km^2^ from 61–90 days, 0.47 km^2^ from 91–120 days, 3.21 km^2^ from 121–150 days, 2.55 km^2^ from 151–180 days, and 3.97 km^2^ from 181–210 days.

We used KDE method to estimate the home range and core activity area of the red panda (Fig 1B). The analysis revealed that the home range was 24.40 km^2^ during 0–30 days post-release and 19.22 km^2^ during 31–60 days. A significant reduction in home range was observed after 60 days: it measured 4.16 km^2^ from 61–90 days, 1.08 km^2^ from 91–120 days, 6.19 km^2^ from 121–150 days, 4.64 km^2^ from 151–180 days, and 7.83 km^2^ from 181–210 days. Similarly, the core activity area exhibited a comparable trend (Fig 1B), being larger during the initial post-release period with values of 4.56 km^2^ for 0–30 days and 5.34 km^2^ for 31–60 days; however, a notable decline occurred thereafter, with values ranging from 0.26 to 1.62 km².

### Habitat selection characteristics after release

#### Altitude.

The altitude of the activity area of the released red panda exhibited significant differences across various time periods after release, with the magnitude of differences markedly decreasing after 60 days. During the 0–30 and 31–60 day periods, the altitude distribution of the released individual significantly differed from that of wild conspecifics. However, after 60 days, specifically during the 61–90, 91–120, 151–180, and 181–210 day periods, no significant differences were observed in altitude utilization between the released and wild individuals. The only exception was noted during the 121–150 day period (Table 1).

**Table 1:** Variations in altitude utilization by a female red panda across different time periods and in comparison with wild individuals. Different capital letters indicated significant differences in red pandas’ utilization of various altitude, whereas identical capital letters suggested no significant differences. All statistical analyses were conducted at a 95% confidence level.

Period Group	Value (m)	Statistical analysis value		
		*df*	Chi-Square	*P*
0-30 d	2115.06 ± 330.33^A^	7	261.91	<0.0001
31-60 d	2943.77 ± 199.15^B^			
61-90 d	2535.43 ± 139.96^C^			
91-120 d	2529.23 ± 70.77^C^			
121-150 d	2993.32 ± 213.59^B^			
151-180 d	2428.14 ± 278.44 ^AC^			
181-210 d	2624.04 ± 349.55^C^			
Wild individuals	2523.20 ± 279.72^C^			

#### Aspect and slope.

In terms of aspect utilization, no significant differences were observed among the time periods of 0–30 days, 31–60 days, 61–90 days, 91–120 days, 121–150 days, 151–180 days, and 180–210 days. Furthermore, no significant differences were detected between the released and wild individuals in aspect utilization (Table 2). In contrast to aspect, significant differences in slope utilization were found between the 0–30 days period and each of the subsequent periods: 31–60 days, 61–90 days, 91–120 days, 121–150 days, 151–180 days, and 180–210 days. However, no statistically significant differences were identified in slope utilization across the six time intervals following the initial 0–30 days period. Similarly, significant differences between the released and wild individuals in slope utilization were observed only during the 0–30 days period (Table 3).

**Table 2:** Variations in aspect utilization by a female red panda across different time periods and in comparison with wild individuals. Different capital letters indicated significant differences in red pandas’ utilization of various aspect, whereas identical capital letters suggested no significant differences. All statistical analyses were conducted at a 95% confidence level.

Period Group	Value (°)	Statistical analysis value		
		*df*	Chi-Square	*P*
0-30 d	160.87 ± 70.23^AB^	7	13.09	0.069
31-60 d	165.23 ± 118.26^AB^			
61-90 d	153.10 ± 135.64^AB^			
91-120 d	206.20 ± 147.57^A^			
121-150 d	191.66 ± 144.77^A^			
151-180 d	122.09 ± 133.92^BC^			
181-210 d	175.40 ± 146.93^AB^			
Wild individuals	137.19 ± 89.35^AB^			

**Table 3:** Variations in slope utilization by a female red panda across different time periods and in comparison with their wild individuals. Different capital letters indicated significant differences in red pandas’ utilization of various slope, whereas identical capital letters suggested no significant differences. All statistical analyses were conducted at a 95% confidence level.

Period Group	Value (°)	Statistical analysis value		
		*df*	Chi-Square	*P*
0-30 d	30.49 ± 11.20^A^	7	56.18	<0.0001
31-60 d	21.60 ± 10.99^B^			
61-90 d	18.55 ± 7.16^B^			
91-120 d	18.74 ± 8.13^B^			
121-150 d	22.30 ± 12.17^B^			
151-180 d	18.54 ± 8.06^B^			
181-210 d	20.79 ± 7.68^B^			
Wild individuals	23.86 ± 10.60^B^			

### Optimal habitat utilization by the red Panda after release

**Fig 2:**
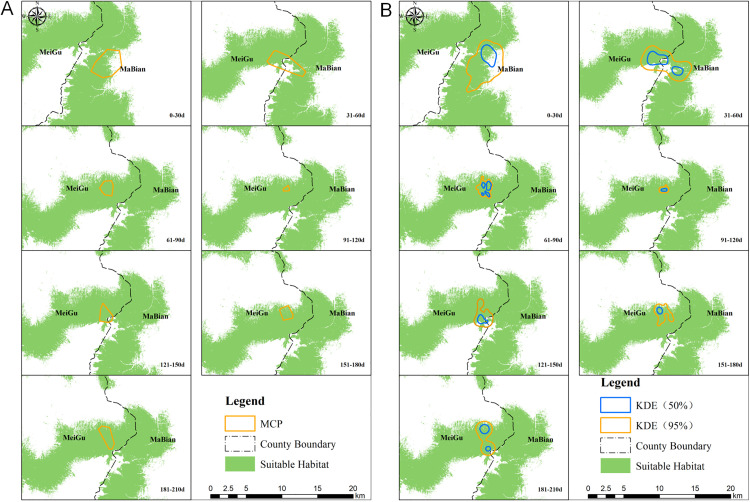
Analysis of spatial overlap between the released red panda and the suitable habitat of local wild individuals. A: Overlap between the activity range of the released red panda, estimated using the minimum convex polygon method, and the suitable habitat of wild individuals. B: Overlap between the activity range and core activity area of the released red panda, estimated using the kernel density estimation method, and the suitable habitat of wild individuals.

The overlap between the home range of the red panda estimated by the MCP method and the suitable habitats for wild individuals indicated that the overlap ratios were 65.76% and 72.48% during the periods of 0–30 days and 31–60 days, respectively; while all subsequent periods post-release periods showed overlap ratios exceeding 90%, specifically: 61–90 days (99.71%), 91–120 days (98.70%), 121–150 days (91.72%), 151–180 days (94.42%), and 181–210 days (98.89%) (Fig 2A).

Similarly, during the periods of 0–30 days, 31–60 days, and 121–150 days post-release, the overlap between home range estimated by KDE method and suitable habitats for wild individuals were 69.17%, 80.10%, and 77.33% respectively. In contrast, the overlap ratios exceeded 90% in other time intervals (61–90 days: 99.51%, 91–120 days: 99.50%, 151–180 days: 91.15%, and 181–210 days: 92.25%) (Fig 2B). Regarding the core activity area, consistent results were observed across the seven time periods following release, indicating that the overlap ratios between the core activity areas of the red panda and suitable habitats of wild individuals were 58.56% for 0–30 days, 84.61% for 31–60 days, and 85.53% for 121–150 days. Additionally, overlap ratios in other time periods exceeded 90% (61–90 days: 99.60%, 91–120 days: 100%, 151–180 days: 94.37%, and 181–210 days: 96.13%) (Fig 2B).

## Discussion

Restrictive living circumstances, such as those in captivity, can induce wild animals to exhibit other abnormal behaviors [41], and may even potentially lead to a lack of fundamental survival skills necessary in the wild [42]. Studies have indicated that giant panda demonstrated unstable habitat selection patterns during the initial phases after release into the wild [35]. The giant and red panda are sympatric species, both of which primarily consume bamboo as their main food source [31]. Our findings suggested that red panda underwent an adaptive process in terms of space utilization and habitat selection following release into the wild. Previous studies have examined the habitat utilization pattern of red panda, observing similar behaviors across different regions; however, it was worth noting that significant regional differences also existed [43]. Nevertheless, the main mechanisms driving habitat selection in red panda has largely been based on qualitative inferences from sporadic field observations, lacking robust quantitative empirical support. Relatively limited research has been conducted on the habitat and space utilization of red panda from a behavioral ecology perspective, resulting in a certain gaps in our understanding of its environmental adaptability. This study included only a single sample, due to the accidental detachment of the GPS collars of the other two individuals. Nevertheless, it represented the first use of GPS collar technology to track red panda and analyzed its space utilization and habitat selection characteristics after release, offering novel insight into environmental adaptation process, although further case studies are needed to confirm the findings.

Outside the breeding season, adult red pandas typically reside solitarily within its respective territories, demarcating their through scent marking [7]. Researchers have used radio collar technology to study the activity range of red pandas. In Fengtongzhai Nature Reserve, their home range was reported as 1.03 km^2^, with the core activity area of 0.26 km^2^; whereas in Wolong Nature Reserve, their home range was 2.20 km^2^, with frequent overlaps between individual home ranges [44,45]. In this study, we used the MCP and KDE methods to estimate the home range of a released red panda. The results showed that its home range was 2.43 km^2^ and 4.78 km^2^, with the core activity area reaching 1.2 km^2^. These findings differ from previous studies, which may be attributed to variations in technology and research areas, including possible biases associated with radio telemetry. Red panda has a relatively narrower activity range compared to other animals and exhibit a stronger preference for habitats with higher suitability [10,46]. The activity area of the released red panda decreased significantly 60 days after release, particularly in the fourth month, when its range was confined to approximately 1 km^2^. This phenomenon was considered as an energy-saving mechanism, reflecting its unique environmental adaptation to a bamboo-based diet [47]. Additionally, previous studies had shown that the MCP method was more susceptible to outliers, with a few specific outliers significantly influencing the estimated home range size. In contrast, the KDE method reduced the impact of outliers and provided a more accurate representation of an animal’s spatial use patterns [13,48]. In our study, the home range estimated using the MCP method was smaller than that estimated with the KDE method. However, the KDE results were consistent with those of previous studies.

Besides genetic factors, the habitat selection of wild animals is also influenced by population density, intraspecific and interspecific relationships, as well as external environmental factors [49]. The habitat selection of arboreal animals constitutes a flexible behavior, wherein forest type, canopy cover, fallen trees, elevation, slope, orientation, and distance from water sources are key variables [50]. Studies have demonstrated that red panda exhibited a preference for habitats at higher altitudes, steep slopes, moderate forest densities, abundant bamboo resources, and proximity to water sources [11,25]. Nevertheless, its habitat preferences vary across regions. For example, red panda residing in the Qionglai Mountains prefered slopes facing south or west with lower forest densities and a greater number of fallen trees and stumps in the bamboo forests. In contrast, red panda in the Daxiangling Mountains favored south-facing slopes where the density of coniferous forests exceeds 80%. Meanwhile, red panda inhabiting the Liangshan Mountains tended to occupy areas with moderate forest densities and close proximity to water sources [47,51]. In Singalila National Park, the relatively high bamboo coverage, bamboo height, and canopy cover were identified as important components of red panda habitats [22]. These differences may be attributed to variations in food availability and geographical conditions across regions, reflecting the environmental adaptation mechanisms employed by red pandas in selecting suitable habitats [10]. Although previous studies have frequently used steep terrain as a common indicator for red panda habitat selection, our study reveals that the released red panda initially moved through areas with steeper slopes and later stabilized in regions with more gentler slopes (approximately 20°). The red panda’s preference for gentler slopes may be related to energy conservation and represent a behavioral adaptation to its environment [52]. Naturally, this selection could also be associated with the distribution of food sources [8]. Notably, the released red panda in this study showed no significant preference for slope orientation during either the initial and later stages, which may be due to the spatial distribution of available resources. Although primarily feeding on bamboo, red pandas exhibit distinct preference for different parts and ages of bamboo [53]. To optimize foraging strategies, red pandas selectively choose feeding sites with higher concentrations of food, thereby maximizing nutrient intake while minimizing energy expenditure during foraging [18].

Over an extended period, comprehensive research on wildlife behavioral ecology has been impeded by multiple challenges. These include the natural wariness of wild animals, which makes direct observation and data collection difficult, as well as the complexity of natural environments that complicate tracking efforts [24]. Additionally, identifying and distinguishing individual and sex characteristics from a macroscopic perspective presents further difficulties [54]. Fortunately, the application of wildlife tracking technologies such as microsatellite molecular markers, automatic infrared cameras, and GPS collar has gradually alleviated these constraints [43]. However, two primary limitations remain when using GPS collar to study animal behavior: first, the battery life of GPS collars is limited, which may not meet the long-term monitoring needs of researchers [55]; second, opportunities to study endangered species using GPS collars are scarce, and collar loss in the wild often result in a small sample size [56,57]. As in our current study, the sample size was limited to one individual, and the monitoring period spanned only seven months. Despite these limitations, we argued that this approach represented a feasible starting point for preliminary research. Nevertheless, we recommend conducting further studies with larger sample sizes to better understand the environmental adaptation mechanisms of red pandas through GPS collar technology. In future ecological studies on wild red pandas, integrating traditional ecological surveys with GPS collar technology will be of critical importance. Moreover, the application of geometric framework modeling techniques fto comprehensively analyze red pandas’ space utilization and other behavioral patterns will enhance our understanding of their adaptation mechanisms across diverse environments. These integrated approaches are expected to elevate red panda ecological research to a new level and deepen our understanding of their adaptive strategies in various environmental contexts.

## Supporting information

S1 Table The locations of the released red panda was recorded using GPS collars, and the corresponding elevation, slope, and aspect values at these locations were extracted using GIS software. (DOCX)

S2 Table The locations of wild red pandas were collected through field surveys in study area, and the corresponding elevation, slope, and aspect values at these locations were extracted using GIS software. (DOCX)
