# Supplementary material for: Study on the environmental adaptation characteristics of red panda release into the wild
Source: PLoS One. 2025 Oct 1;20(10):e0331776. doi: 10.1371/journal.pone.0331776 (PMC12488013; doi:10.1371/journal.pone.0331776)
Supplement: S1 Table — (DOCX) [file pone.0331776.s001.docx]

**S1 Table. The locations of the released red panda was recorded using GPS collars, and the corresponding elevation, slope, and aspect values at these locations were extracted using GIS software.**

| **Period Group** | **Latitude (°)** | **Longitude (°)** | **Altitude (m)** | **Aspect (°)** | **Slope (°)** |
| --- | --- | --- | --- | --- | --- |
| 0~30d | 28.69286 | 103.35997 | 2167 | 116.57 | 17.13 |
|  | 28.69153 | 103.35911 | 2245 | 65.02 | 35.76 |
|  | 28.69028 | 103.36014 | 2170 | 118.54 | 38.28 |
|  | 28.65858 | 103.33600 | 2248 | 160.50 | 47.86 |
|  | 28.67647 | 103.35958 | 1756 | 218.40 | 34.93 |
|  | 28.66871 | 103.35795 | 1468 | 78.89 | 53.82 |
|  | 28.66276 | 103.36096 | 1467 | 56.56 | 27.25 |
|  | 28.65035 | 103.37274 | 1602 | 291.50 | 23.33 |
|  | 28.64889 | 103.37538 | 1753 | 240.46 | 29.21 |
|  | 28.64703 | 103.37102 | 1589 | 23.32 | 44.81 |
|  | 28.64813 | 103.35246 | 1356 | 176.06 | 25.24 |
|  | 28.64907 | 103.32903 | 2172 | 62.97 | 12.57 |
|  | 28.64257 | 103.31870 | 2234 | 72.05 | 31.18 |
|  | 28.68859 | 103.35360 | 2129 | 136.83 | 28.33 |
|  | 28.69023 | 103.35429 | 2147 | 136.27 | 27.30 |
|  | 28.68530 | 103.35532 | 1991 | 139.60 | 26.58 |
|  | 28.68819 | 103.35988 | 2102 | 159.83 | 22.94 |
|  | 28.68431 | 103.35057 | 2053 | 88.70 | 35.52 |
|  | 28.68073 | 103.35756 | 1971 | 149.62 | 7.76 |
|  | 28.68151 | 103.35296 | 1886 | 93.01 | 34.77 |
|  | 28.68408 | 103.35920 | 1881 | 159.88 | 23.56 |
|  | 28.69083 | 103.34796 | 2349 | 112.10 | 49.33 |
|  | 28.68041 | 103.34711 | 1882 | 150.46 | 15.62 |
|  | 28.67627 | 103.35053 | 1755 | 168.81 | 56.02 |
|  | 28.68897 | 103.35012 | 2145 | 92.52 | 32.83 |
|  | 28.67616 | 103.35261 | 1763 | 148.50 | 30.52 |
|  | 28.69041 | 103.34466 | 2373 | 253.96 | 44.51 |
|  | 28.68999 | 103.34449 | 2337 | 249.85 | 43.27 |
|  | 28.69034 | 103.34533 | 2414 | 229.97 | 27.90 |
|  | 28.69006 | 103.34542 | 2404 | 223.90 | 30.81 |
|  | 28.63723 | 103.32544 | 1886 | 146.48 | 37.89 |
|  | 28.69024 | 103.34521 | 2414 | 229.97 | 27.90 |
|  | 28.69034 | 103.34590 | 2439 | 210.02 | 22.85 |
|  | 28.69011 | 103.34601 | 2429 | 188.13 | 18.98 |
|  | 28.66071 | 103.34917 | 1985 | 52.13 | 29.02 |
|  | 28.67294 | 103.36320 | 1591 | 149.04 | 5.40 |
|  | 28.64891 | 103.37137 | 1504 | 270.00 | 26.32 |
|  | 28.65463 | 103.36423 | 1209 | 65.12 | 31.66 |
|  | 28.66340 | 103.33755 | 2511 | 57.31 | 17.89 |
|  | 28.65208 | 103.32731 | 2279 | 117.35 | 21.66 |
|  | 28.68962 | 103.34526 | 2388 | 221.72 | 35.02 |
|  | 28.68935 | 103.34564 | 2388 | 230.62 | 35.10 |
|  | 28.68989 | 103.34523 | 2404 | 223.90 | 30.81 |
|  | 28.69021 | 103.34647 | 2441 | 183.53 | 18.21 |
|  | 28.64548 | 103.34555 | 1446 | 127.73 | 15.20 |
|  | 28.65036 | 103.31801 | 1952 | 45.92 | 19.57 |
|  | 28.64455 | 103.33092 | 1729 | 159.86 | 48.20 |
|  | 28.63783 | 103.32645 | 1908 | 165.02 | 30.79 |
|  | 28.65281 | 103.31672 | 2022 | 35.67 | 22.98 |
|  | 28.67846 | 103.33179 | 2346 | 158.79 | 30.23 |
|  | 28.67394 | 103.33385 | 2193 | 58.47 | 39.93 |
|  | 28.68465 | 103.33953 | 2172 | 160.00 | 50.68 |
|  | 28.67787 | 103.34143 | 1991 | 114.81 | 27.99 |
|  | 28.69430 | 103.34221 | 2527 | 38.16 | 8.21 |
|  | 28.69037 | 103.34508 | 2402 | 244.83 | 36.63 |
|  | 28.69010 | 103.34457 | 2337 | 249.85 | 43.27 |
|  | 28.69059 | 103.34405 | 2320 | 240.72 | 38.80 |
|  | 28.69041 | 103.34466 | 2373 | 253.96 | 44.51 |
|  | 28.69011 | 103.34601 | 2429 | 188.13 | 18.98 |
|  | 28.69034 | 103.34533 | 2414 | 229.97 | 27.90 |
|  | 28.69006 | 103.34542 | 2404 | 223.90 | 30.81 |
|  | 28.69024 | 103.34521 | 2414 | 229.97 | 27.90 |
|  | 28.69418 | 103.34246 | 2523 | 70.64 | 9.03 |
|  | 28.69034 | 103.34590 | 2439 | 210.02 | 22.85 |
|  | 28.68962 | 103.34526 | 2388 | 221.72 | 35.02 |
|  | 28.69103 | 103.34413 | 2362 | 222.96 | 43.94 |
|  | 28.69076 | 103.34426 | 2342 | 224.62 | 41.07 |
|  | 28.69085 | 103.34406 | 2342 | 224.62 | 41.07 |
|  | 28.69072 | 103.34372 | 2307 | 225.60 | 28.83 |
|  | 28.69057 | 103.34355 | 2295 | 215.02 | 32.16 |
| 31~60d | 28.69309 | 103.34039 | 2536 | 160.29 | 29.99 |
|  | 28.69316 | 103.33764 | 2647 | 122.54 | 29.15 |
|  | 28.69339 | 103.33884 | 2566 | 131.95 | 40.74 |
|  | 28.69187 | 103.32989 | 2976 | 290.46 | 16.17 |
|  | 28.69096 | 103.33084 | 2957 | 136.05 | 43.24 |
|  | 28.68705 | 103.33506 | 2472 | 148.26 | 39.51 |
|  | 28.68705 | 103.33385 | 2544 | 139.32 | 31.36 |
|  | 28.68464 | 103.33179 | 2551 | 87.17 | 39.35 |
|  | 28.68109 | 103.33084 | 2570 | 115.75 | 20.94 |
|  | 28.68302 | 103.33075 | 2636 | 86.78 | 29.97 |
|  | 28.68304 | 103.32318 | 2710 | 88.75 | 29.05 |
|  | 28.68432 | 103.32477 | 2836 | 197.64 | 47.99 |
|  | 28.68959 | 103.32154 | 3132 | 359.03 | 13.45 |
|  | 28.68903 | 103.32180 | 3134 | 162.20 | 24.90 |
|  | 28.69057 | 103.32223 | 3091 | 335.46 | 37.63 |
|  | 28.68744 | 103.32258 | 3021 | 153.07 | 29.81 |
|  | 28.68696 | 103.32559 | 3030 | 179.50 | 25.00 |
|  | 28.69163 | 103.32606 | 2937 | 46.23 | 38.76 |
|  | 28.69136 | 103.32142 | 3023 | 0.71 | 33.30 |
|  | 28.69569 | 103.31784 | 3103 | 118.58 | 33.80 |
|  | 28.69612 | 103.31044 | 3218 | 13.30 | 12.90 |
|  | 28.69563 | 103.30988 | 3251 | 14.86 | 11.61 |
|  | 28.69541 | 103.31053 | 3246 | 14.74 | 9.05 |
|  | 28.70101 | 103.30623 | 3155 | 353.80 | 20.56 |
|  | 28.69204 | 103.30132 | 3177 | 282.38 | 18.80 |
|  | 28.70544 | 103.29612 | 2932 | 301.46 | 17.66 |
|  | 28.70453 | 103.29289 | 2796 | 62.28 | 15.12 |
|  | 28.69869 | 103.29052 | 3020 | 5.91 | 13.30 |
|  | 28.69583 | 103.28954 | 3051 | 287.65 | 10.60 |
|  | 28.69689 | 103.29362 | 3083 | 0.44 | 27.61 |
|  | 28.69987 | 103.30012 | 3109 | 244.87 | 26.84 |
|  | 28.69588 | 103.28545 | 3023 | 65.56 | 5.60 |
|  | 28.69978 | 103.28932 | 3023 | 44.25 | 23.55 |
|  | 28.70020 | 103.29091 | 2980 | 309.34 | 32.60 |
|  | 28.70043 | 103.29650 | 2931 | 258.83 | 32.97 |
|  | 28.70490 | 103.28541 | 2898 | 79.08 | 13.24 |
|  | 28.70534 | 103.28726 | 2897 | 324.02 | 20.09 |
|  | 28.70741 | 103.28638 | 2819 | 66.80 | 17.16 |
|  | 28.70549 | 103.29044 | 2874 | 85.71 | 18.02 |
|  | 28.70875 | 103.29126 | 2777 | 21.61 | 13.01 |
|  | 28.70556 | 103.30145 | 3046 | 338.43 | 29.35 |
|  | 28.70290 | 103.30614 | 3135 | 244.65 | 4.87 |
|  | 28.70320 | 103.30971 | 3135 | 38.66 | 5.93 |
|  | 28.70166 | 103.30928 | 3095 | 150.42 | 9.79 |
|  | 28.70516 | 103.31066 | 3138 | 303.37 | 11.26 |
|  | 28.70708 | 103.30907 | 3070 | 319.16 | 17.55 |
|  | 28.70478 | 103.30739 | 3118 | 241.39 | 5.80 |
|  | 28.70376 | 103.30511 | 3116 | 304.88 | 9.26 |
|  | 28.70210 | 103.30498 | 3127 | 92.73 | 4.87 |
|  | 28.70217 | 103.29977 | 3108 | 295.68 | 30.51 |
|  | 28.69998 | 103.29857 | 3028 | 266.82 | 23.68 |
|  | 28.70213 | 103.29569 | 2965 | 267.92 | 24.05 |
|  | 28.69970 | 103.27922 | 2923 | 101.98 | 7.79 |
|  | 28.70728 | 103.29435 | 2851 | 280.31 | 24.38 |
|  | 28.71029 | 103.29220 | 2719 | 42.06 | 24.12 |
|  | 28.70884 | 103.28853 | 2793 | 3.50 | 21.70 |
|  | 28.69593 | 103.28330 | 3047 | 358.21 | 14.55 |
|  | 28.71257 | 103.28863 | 2777 | 328.33 | 12.62 |
|  | 28.70098 | 103.28403 | 2969 | 23.75 | 12.49 |
|  | 28.71341 | 103.29192 | 2734 | 14.93 | 7.17 |
| 61~90d | 28.71222 | 103.29366 | 2702 | 300.96 | 10.71 |
|  | 28.71112 | 103.29315 | 2702 | 329.42 | 11.71 |
|  | 28.71203 | 103.29229 | 2737 | 143.23 | 25.68 |
|  | 28.71349 | 103.29087 | 2738 | 20.56 | 7.89 |
|  | 28.71481 | 103.28967 | 2719 | 198.44 | 1.47 |
|  | 28.71429 | 103.29225 | 2718 | 51.98 | 15.80 |
|  | 28.71621 | 103.29182 | 2700 | 0.00 | 3.25 |
|  | 28.71326 | 103.28463 | 2763 | 37.53 | 19.43 |
|  | 28.71315 | 103.28356 | 2764 | 313.83 | 15.69 |
|  | 28.71428 | 103.28386 | 2737 | 35.31 | 13.41 |
|  | 28.71353 | 103.28721 | 2738 | 65.56 | 8.36 |
|  | 28.71525 | 103.28188 | 2702 | 7.59 | 20.21 |
|  | 28.71664 | 103.27969 | 2645 | 111.55 | 27.42 |
|  | 28.71770 | 103.28253 | 2628 | 357.58 | 29.95 |
|  | 28.71860 | 103.27999 | 2644 | 74.58 | 13.71 |
|  | 28.72109 | 103.28016 | 2598 | 61.91 | 31.43 |
|  | 28.72297 | 103.27741 | 2585 | 32.47 | 11.94 |
|  | 28.72176 | 103.27573 | 2592 | 264.81 | 15.04 |
|  | 28.72444 | 103.28008 | 2541 | 32.15 | 18.53 |
|  | 28.72045 | 103.28472 | 2639 | 301.76 | 16.72 |
|  | 28.72211 | 103.28584 | 2559 | 354.21 | 15.70 |
|  | 28.72478 | 103.28528 | 2496 | 38.02 | 15.80 |
|  | 28.72301 | 103.28390 | 2555 | 329.68 | 26.47 |
|  | 28.72512 | 103.28257 | 2483 | 6.47 | 21.59 |
|  | 28.72651 | 103.28304 | 2443 | 67.38 | 6.02 |
|  | 28.72825 | 103.28395 | 2376 | 337.17 | 18.49 |
|  | 28.73035 | 103.28227 | 2363 | 41.70 | 16.63 |
|  | 28.72851 | 103.28584 | 2359 | 25.50 | 16.28 |
|  | 28.72543 | 103.28717 | 2436 | 62.39 | 26.50 |
|  | 28.72614 | 103.28562 | 2458 | 357.05 | 21.49 |
|  | 28.73500 | 103.28307 | 2243 | 64.60 | 23.95 |
|  | 28.72512 | 103.29504 | 2460 | 290.82 | 17.12 |
|  | 28.72613 | 103.29500 | 2450 | 294.35 | 22.92 |
|  | 28.73116 | 103.29244 | 2276 | 271.12 | 22.47 |
|  | 28.72899 | 103.29083 | 2363 | 68.43 | 29.35 |
|  | 28.72679 | 103.28988 | 2392 | 3.81 | 13.70 |
|  | 28.72530 | 103.29192 | 2426 | 43.41 | 22.44 |
|  | 28.72325 | 103.29089 | 2443 | 286.43 | 30.43 |
|  | 28.72628 | 103.29319 | 2388 | 267.71 | 16.92 |
|  | 28.72620 | 103.29162 | 2394 | 64.32 | 25.07 |
|  | 28.72146 | 103.29278 | 2548 | 344.85 | 21.96 |
|  | 28.72242 | 103.29061 | 2458 | 327.30 | 24.59 |
|  | 28.72432 | 103.29203 | 2442 | 40.40 | 26.58 |
|  | 28.72553 | 103.29276 | 2411 | 336.28 | 34.81 |
|  | 28.72713 | 103.29300 | 2408 | 252.18 | 13.41 |
|  | 28.72786 | 103.29124 | 2375 | 57.31 | 17.89 |
|  | 28.72879 | 103.29304 | 2361 | 298.22 | 20.67 |
|  | 28.72584 | 103.29351 | 2411 | 329.62 | 15.25 |
|  | 28.72295 | 103.29276 | 2499 | 29.54 | 15.62 |
|  | 28.71984 | 103.29089 | 2623 | 5.30 | 21.55 |
|  | 28.71803 | 103.28803 | 2669 | 37.73 | 15.20 |
|  | 28.71586 | 103.28801 | 2719 | 324.46 | 7.94 |
|  | 28.71755 | 103.28627 | 2665 | 324.62 | 18.94 |
|  | 28.72057 | 103.28936 | 2574 | 5.03 | 26.96 |
|  | 28.72137 | 103.29491 | 2500 | 34.22 | 13.77 |
|  | 28.71975 | 103.29330 | 2547 | 81.19 | 16.24 |
|  | 28.72218 | 103.29257 | 2516 | 18.14 | 26.33 |
|  | 28.72621 | 103.29257 | 2374 | 17.65 | 10.60 |
| 91~120d | 28.72239 | 103.29470 | 2491 | 293.96 | 9.07 |
|  | 28.72203 | 103.29472 | 2490 | 48.90 | 14.19 |
|  | 28.72242 | 103.29251 | 2510 | 32.62 | 13.53 |
|  | 28.72211 | 103.29256 | 2516 | 18.14 | 26.33 |
|  | 28.72441 | 103.29478 | 2461 | 307.57 | 14.89 |
|  | 28.72488 | 103.29715 | 2493 | 328.84 | 38.35 |
|  | 28.72570 | 103.29754 | 2465 | 313.45 | 32.48 |
|  | 28.72528 | 103.29884 | 2521 | 353.90 | 22.78 |
|  | 28.72565 | 103.30097 | 2484 | 354.13 | 23.56 |
|  | 28.72534 | 103.30075 | 2498 | 349.27 | 32.26 |
|  | 28.72479 | 103.30098 | 2533 | 340.77 | 10.46 |
|  | 28.72411 | 103.29999 | 2542 | 350.13 | 20.74 |
|  | 28.72350 | 103.29915 | 2567 | 49.76 | 11.70 |
|  | 28.72232 | 103.29969 | 2628 | 356.07 | 28.03 |
|  | 28.72296 | 103.29902 | 2576 | 58.50 | 16.43 |
|  | 28.72378 | 103.29866 | 2579 | 30.07 | 5.09 |
|  | 28.72263 | 103.29758 | 2556 | 326.94 | 16.95 |
|  | 28.72298 | 103.29734 | 2546 | 352.24 | 10.21 |
|  | 28.72277 | 103.29714 | 2552 | 0.00 | 8.76 |
|  | 28.71405 | 103.28579 | 2721 | 355.16 | 13.50 |
|  | 28.72334 | 103.29875 | 2576 | 81.53 | 10.90 |
|  | 28.72250 | 103.29926 | 2598 | 4.01 | 32.54 |
|  | 28.72389 | 103.29667 | 2535 | 323.30 | 15.54 |
|  | 28.72415 | 103.29515 | 2487 | 322.06 | 31.24 |
|  | 28.72322 | 103.29241 | 2499 | 31.43 | 9.71 |
|  | 28.72312 | 103.29695 | 2555 | 43.83 | 15.69 |
|  | 28.72404 | 103.29767 | 2557 | 295.13 | 26.84 |
|  | 28.72338 | 103.29670 | 2550 | 11.82 | 19.61 |
|  | 28.72432 | 103.29601 | 2507 | 333.44 | 20.84 |
|  | 28.72444 | 103.29231 | 2434 | 31.55 | 15.17 |
|  | 28.72530 | 103.29287 | 2427 | 327.36 | 23.19 |
|  | 28.72476 | 103.29297 | 2444 | 299.90 | 20.51 |
|  | 28.72371 | 103.29373 | 2457 | 57.80 | 7.37 |
|  | 28.72246 | 103.29435 | 2484 | 332.97 | 12.57 |
|  | 28.72267 | 103.29472 | 2486 | 288.95 | 16.02 |
|  | 28.72273 | 103.29447 | 2480 | 311.73 | 11.36 |
|  | 28.72931 | 103.29964 | 2369 | 34.95 | 24.70 |
|  | 28.72339 | 103.29714 | 2542 | 331.99 | 12.18 |
|  | 28.72288 | 103.29985 | 2586 | 325.19 | 27.40 |
|  | 28.72433 | 103.30089 | 2540 | 6.34 | 10.40 |
|  | 28.72279 | 103.30138 | 2573 | 52.91 | 22.62 |
|  | 28.72308 | 103.30138 | 2563 | 55.37 | 25.54 |
|  | 28.71334 | 103.28421 | 2769 | 342.53 | 24.66 |
| 121~150d | 28.73146 | 103.29081 | 2310 | 40.66 | 24.43 |
|  | 28.72794 | 103.27624 | 2482 | 335.23 | 13.07 |
|  | 28.72059 | 103.29438 | 2524 | -1.00 | 0.00 |
|  | 28.72078 | 103.29434 | 2524 | 106.07 | 13.98 |
|  | 28.72067 | 103.29457 | 2524 | -1.00 | 0.00 |
|  | 28.72073 | 103.29428 | 2533 | 130.71 | 24.70 |
|  | 28.72006 | 103.29378 | 2525 | 53.13 | 13.67 |
|  | 28.71227 | 103.29085 | 2757 | 327.10 | 4.69 |
|  | 28.69851 | 103.28963 | 3017 | 356.42 | 7.41 |
|  | 28.69862 | 103.28986 | 3017 | 356.42 | 7.41 |
|  | 28.69847 | 103.28988 | 3020 | 349.29 | 8.68 |
|  | 28.69832 | 103.29027 | 3024 | 348.69 | 9.39 |
|  | 28.69798 | 103.29072 | 3030 | 335.56 | 13.76 |
|  | 28.69880 | 103.29517 | 3030 | 12.38 | 34.24 |
|  | 28.69875 | 103.29516 | 3045 | 14.93 | 20.69 |
|  | 28.70022 | 103.29479 | 2918 | 2.52 | 32.83 |
|  | 28.69876 | 103.29519 | 3030 | 12.38 | 34.24 |
|  | 28.69871 | 103.29501 | 3047 | 1.15 | 22.07 |
|  | 28.69871 | 103.29514 | 3045 | 14.93 | 20.69 |
|  | 28.69893 | 103.29508 | 3033 | 2.92 | 29.08 |
|  | 28.69889 | 103.29527 | 3030 | 12.38 | 34.24 |
|  | 28.69828 | 103.29307 | 3048 | 7.22 | 16.18 |
|  | 28.69888 | 103.29535 | 3030 | 12.38 | 34.24 |
|  | 28.69874 | 103.29515 | 3045 | 14.93 | 20.69 |
|  | 28.69878 | 103.29530 | 3030 | 12.38 | 34.24 |
|  | 28.70261 | 103.29417 | 2839 | 284.97 | 35.82 |
|  | 28.70308 | 103.29402 | 2810 | 282.36 | 31.21 |
|  | 28.70863 | 103.30380 | 2994 | 301.81 | 20.65 |
|  | 28.70321 | 103.29559 | 2934 | 283.74 | 29.40 |
|  | 28.70113 | 103.29428 | 2883 | 351.61 | 14.03 |
|  | 28.70127 | 103.29434 | 2881 | 271.12 | 11.68 |
|  | 28.69882 | 103.29553 | 3028 | 29.10 | 41.99 |
|  | 28.70330 | 103.29411 | 2844 | 260.19 | 47.58 |
|  | 28.70522 | 103.29582 | 2924 | 301.50 | 23.86 |
|  | 28.70497 | 103.29380 | 2862 | 264.69 | 35.00 |
|  | 28.70386 | 103.30896 | 3133 | 43.26 | 10.72 |
|  | 28.69883 | 103.29513 | 3033 | 2.92 | 29.08 |
|  | 28.69894 | 103.29506 | 3033 | 2.92 | 29.08 |
|  | 28.69892 | 103.29533 | 3030 | 12.38 | 34.24 |
|  | 28.69880 | 103.29521 | 3030 | 12.38 | 34.24 |
|  | 28.69882 | 103.29535 | 3030 | 12.38 | 34.24 |
|  | 28.69882 | 103.29561 | 3028 | 29.10 | 41.99 |
|  | 28.71027 | 103.30318 | 2913 | 327.65 | 16.06 |
|  | 28.70270 | 103.29430 | 2839 | 273.24 | 40.72 |
|  | 28.70678 | 103.29389 | 2815 | 260.61 | 27.56 |
|  | 28.68657 | 103.26482 | 3162 | 9.64 | 12.29 |
|  | 28.69904 | 103.29594 | 2973 | 31.72 | 40.31 |
|  | 28.69885 | 103.29679 | 2968 | 348.41 | 17.89 |
|  | 28.70087 | 103.29659 | 2962 | 256.41 | 32.54 |
|  | 28.70301 | 103.29411 | 2844 | 270.23 | 45.73 |
|  | 28.66670 | 103.29482 | 3115 | 178.11 | 20.26 |
|  | 28.70322 | 103.29384 | 2817 | 265.91 | 38.54 |
|  | 28.70086 | 103.29655 | 2962 | 256.41 | 32.54 |
|  | 28.70042 | 103.29721 | 2985 | 225.47 | 34.97 |
|  | 28.69978 | 103.29794 | 2998 | 286.34 | 26.11 |
|  | 28.69745 | 103.30172 | 3182 | 325.78 | 20.19 |
|  | 28.69782 | 103.30208 | 3174 | 335.56 | 18.93 |
|  | 28.66660 | 103.27190 | 3491 | 260.54 | 2.82 |
|  | 28.70441 | 103.30919 | 3118 | 67.62 | 8.48 |
|  | 28.69642 | 103.30292 | 3229 | 341.57 | 12.99 |
|  | 28.69634 | 103.30286 | 3229 | 341.57 | 12.99 |
|  | 28.67885 | 103.30344 | 3314 | 203.35 | 38.94 |
|  | 28.69626 | 103.30276 | 3229 | 341.57 | 12.99 |
|  | 28.69656 | 103.30244 | 3215 | 329.59 | 23.39 |
|  | 28.69656 | 103.30298 | 3224 | 336.04 | 9.07 |
|  | 28.69720 | 103.30204 | 3187 | 336.25 | 12.49 |
|  | 28.69777 | 103.30152 | 3152 | 315.00 | 30.56 |
|  | 28.69771 | 103.30142 | 3152 | 315.00 | 30.56 |
|  | 28.69839 | 103.30684 | 3224 | 348.69 | 7.07 |
|  | 28.69858 | 103.30691 | 3221 | 339.62 | 8.61 |
|  | 28.69915 | 103.30700 | 3209 | 13.78 | 12.47 |
|  | 28.69854 | 103.30700 | 3221 | 339.62 | 8.61 |
|  | 28.69845 | 103.30687 | 3224 | 348.69 | 7.07 |
|  | 28.69839 | 103.30696 | 3224 | 348.69 | 7.07 |
| 151~180d | 28.66457 | 103.29648 | 2993 | 195.67 | 30.34 |
|  | 28.70353 | 103.30499 | 3119 | 334.09 | 8.96 |
|  | 28.69456 | 103.29346 | 3130 | 334.80 | 8.66 |
|  | 28.69323 | 103.29796 | 3144 | 349.16 | 21.21 |
|  | 28.64807 | 103.29161 | 3143 | 166.33 | 42.81 |
|  | 28.67373 | 103.31036 | 3093 | 170.10 | 37.88 |
|  | 28.73309 | 103.28754 | 2248 | 356.74 | 26.54 |
|  | 28.73308 | 103.28709 | 2246 | 5.98 | 23.17 |
|  | 28.73306 | 103.28747 | 2248 | 356.74 | 26.54 |
|  | 28.73401 | 103.28859 | 2240 | 6.07 | 10.85 |
|  | 28.73419 | 103.28864 | 2238 | 12.26 | 5.45 |
|  | 28.69971 | 103.29146 | 3024 | 351.57 | 12.48 |
|  | 28.73453 | 103.28870 | 2231 | 357.61 | 11.02 |
|  | 28.73492 | 103.28893 | 2223 | 3.12 | 12.59 |
|  | 28.73492 | 103.28875 | 2223 | 3.12 | 12.59 |
|  | 28.73273 | 103.28681 | 2262 | 342.90 | 18.31 |
|  | 28.73107 | 103.28711 | 2323 | 357.40 | 10.12 |
|  | 28.73490 | 103.28887 | 2223 | 3.12 | 12.59 |
|  | 28.70324 | 103.30593 | 3131 | 319.76 | 3.95 |
|  | 28.73103 | 103.29121 | 2309 | 41.11 | 30.62 |
|  | 28.73112 | 103.29120 | 2309 | 41.11 | 30.62 |
|  | 28.73280 | 103.29525 | 2281 | 323.47 | 15.24 |
|  | 28.73159 | 103.29020 | 2305 | 359.38 | 20.66 |
|  | 28.73848 | 103.29876 | 2123 | 20.04 | 20.14 |
|  | 28.73102 | 103.29116 | 2309 | 41.11 | 30.62 |
|  | 28.73021 | 103.29115 | 2336 | 76.61 | 14.71 |
|  | 28.73167 | 103.29855 | 2294 | 359.19 | 16.06 |
|  | 28.73158 | 103.29140 | 2278 | 54.95 | 18.36 |
|  | 28.73160 | 103.29153 | 2269 | 48.07 | 17.82 |
|  | 28.73212 | 103.29079 | 2276 | 12.53 | 20.49 |
|  | 28.73251 | 103.29053 | 2271 | 59.04 | 17.08 |
|  | 28.73191 | 103.29073 | 2290 | 38.29 | 21.43 |
|  | 28.73114 | 103.29033 | 2326 | 27.98 | 16.37 |
|  | 28.72699 | 103.30601 | 2459 | 10.43 | 27.26 |
|  | 28.73055 | 103.29117 | 2331 | 47.16 | 16.91 |
|  | 28.73095 | 103.29119 | 2323 | 37.27 | 25.11 |
|  | 28.73002 | 103.29119 | 2334 | 93.24 | 23.29 |
|  | 28.73021 | 103.29086 | 2338 | 48.18 | 11.68 |
|  | 28.73491 | 103.28889 | 2223 | 3.12 | 12.59 |
|  | 28.73303 | 103.28932 | 2262 | 26.57 | 6.21 |
|  | 28.73064 | 103.28931 | 2339 | 290.03 | 24.78 |
|  | 28.73018 | 103.29097 | 2338 | 48.18 | 11.68 |
|  | 28.73016 | 103.29083 | 2338 | 48.18 | 11.68 |
|  | 28.73045 | 103.29106 | 2331 | 47.16 | 16.91 |
|  | 28.71497 | 103.28926 | 2715 | 255.58 | 8.33 |
|  | 28.73020 | 103.29100 | 2336 | 76.61 | 14.71 |
|  | 28.73022 | 103.29102 | 2336 | 76.61 | 14.71 |
|  | 28.72947 | 103.29043 | 2362 | 40.91 | 17.84 |
|  | 28.73138 | 103.30167 | 2312 | 302.83 | 16.65 |
|  | 28.72183 | 103.29317 | 2529 | 12.76 | 23.78 |
|  | 28.72154 | 103.29323 | 2541 | 11.31 | 27.33 |
|  | 28.72174 | 103.29305 | 2543 | 7.19 | 22.83 |
|  | 28.71977 | 103.29234 | 2589 | 88.45 | 24.24 |
|  | 28.72076 | 103.30821 | 2641 | 31.33 | 33.22 |
|  | 28.72076 | 103.29435 | 2524 | 106.07 | 13.98 |
|  | 28.72348 | 103.29355 | 2457 | 57.80 | 7.37 |
|  | 28.73310 | 103.29373 | 2269 | 22.07 | 9.19 |
|  | 28.73210 | 103.29081 | 2276 | 12.53 | 20.49 |
|  | 28.73244 | 103.29040 | 2283 | 38.05 | 13.32 |
|  | 28.73289 | 103.29368 | 2276 | 23.81 | 16.77 |
|  | 28.72266 | 103.30534 | 2575 | 311.27 | 14.80 |
|  | 28.73200 | 103.29076 | 2290 | 38.29 | 21.43 |
|  | 28.73379 | 103.29102 | 2244 | 35.44 | 28.91 |
|  | 28.73425 | 103.29099 | 2229 | 36.19 | 22.39 |
| 181~210d | 28.70015 | 103.31851 | 2925 | 57.09 | 46.86 |
|  | 28.72775 | 103.29394 | 2402 | 23.50 | 11.49 |
|  | 28.72936 | 103.29288 | 2345 | 296.37 | 27.49 |
|  | 28.72917 | 103.29293 | 2358 | 305.34 | 15.29 |
|  | 28.72920 | 103.29281 | 2347 | 278.00 | 26.35 |
|  | 28.72910 | 103.29321 | 2359 | 334.50 | 16.28 |
|  | 28.73190 | 103.29095 | 2290 | 38.29 | 21.43 |
|  | 28.73148 | 103.29115 | 2294 | 46.12 | 30.32 |
|  | 28.72907 | 103.29327 | 2359 | 334.50 | 16.28 |
|  | 28.72910 | 103.29319 | 2358 | 305.34 | 15.29 |
|  | 28.73985 | 103.30064 | 2079 | 359.41 | 21.47 |
|  | 28.73070 | 103.29403 | 2325 | 39.09 | 9.49 |
|  | 28.73086 | 103.29426 | 2325 | 39.09 | 9.49 |
|  | 28.73066 | 103.29425 | 2327 | 46.91 | 9.76 |
|  | 28.73062 | 103.29440 | 2316 | 41.31 | 10.10 |
|  | 28.72912 | 103.29324 | 2359 | 334.50 | 16.28 |
|  | 28.71165 | 103.29449 | 2741 | 255.26 | 21.71 |
|  | 28.73272 | 103.29356 | 2276 | 23.81 | 16.77 |
|  | 28.73220 | 103.29402 | 2294 | 33.23 | 15.70 |
|  | 28.73204 | 103.29656 | 2294 | 343.03 | 26.57 |
|  | 28.72979 | 103.29521 | 2345 | 3.29 | 19.46 |
|  | 28.73252 | 103.28753 | 2276 | 1.97 | 25.20 |
|  | 28.73393 | 103.28509 | 2249 | 29.18 | 19.67 |
|  | 28.72958 | 103.28032 | 2416 | 9.78 | 19.69 |
|  | 28.72983 | 103.28848 | 2332 | 50.39 | 16.97 |
|  | 28.71917 | 103.28825 | 2643 | 22.38 | 16.60 |
|  | 28.71914 | 103.29066 | 2644 | 349.05 | 14.36 |
|  | 28.73753 | 103.26981 | 2251 | 9.06 | 15.82 |
|  | 28.71920 | 103.28810 | 2646 | 15.64 | 17.52 |
|  | 28.70488 | 103.29610 | 2938 | 308.23 | 18.81 |
|  | 28.70478 | 103.29656 | 2974 | 288.15 | 26.92 |
|  | 28.70473 | 103.29603 | 2946 | 315.00 | 21.58 |
|  | 28.70475 | 103.29601 | 2946 | 315.00 | 21.58 |
|  | 28.69454 | 103.29378 | 3126 | 101.31 | 11.68 |
|  | 28.70455 | 103.29999 | 3088 | 330.26 | 11.10 |
|  | 28.70562 | 103.30088 | 3041 | 5.19 | 30.20 |
|  | 28.70561 | 103.30077 | 3041 | 5.19 | 30.20 |
|  | 28.70565 | 103.30069 | 3041 | 350.41 | 30.28 |
|  | 28.71177 | 103.30350 | 2913 | 291.04 | 15.77 |
|  | 28.70509 | 103.29948 | 3062 | 331.11 | 28.24 |
|  | 28.69722 | 103.31636 | 3114 | 83.48 | 29.74 |
|  | 28.70507 | 103.29996 | 3071 | 344.66 | 25.41 |
|  | 28.70435 | 103.29128 | 2873 | 18.11 | 24.53 |
|  | 28.68826 | 103.30358 | 3337 | 322.13 | 31.00 |
|  | 28.70484 | 103.30002 | 3081 | 345.11 | 18.33 |
|  | 28.70293 | 103.29549 | 2939 | 288.44 | 31.07 |
