# Supplementary material for: Study on the environmental adaptation characteristics of red panda release into the wild
Source: PLoS One. 2025 Oct 1;20(10):e0331776. doi: 10.1371/journal.pone.0331776 (PMC12488013; doi:10.1371/journal.pone.0331776)
Supplement: S2 Table — (DOCX) [file pone.0331776.s002.docx]

**S2 Table. The locations of wild red pandas were collected through field surveys in study area, and the corresponding elevation, slope, and aspect values at these locations were extracted using GIS software.**

| **Latitude (°)** | **Longitude (°)** | **Altitude (m)** | **Aspect (°)** | **Slope (°)** |
| --- | --- | --- | --- | --- |
| 28.71500 | 103.38767 | 2350 | 32.47 | 20.30 |
| 28.70399 | 103.37341 | 2083 | 182.22 | 36.29 |
| 28.70233 | 103.35928 | 2466 | 36.81 | 35.91 |
| 28.69269 | 103.35168 | 2353 | 124.70 | 10.89 |
| 28.69699 | 103.34761 | 2559 | 78.86 | 28.61 |
| 28.70454 | 103.34271 | 2368 | 274.09 | 18.85 |
| 28.71246 | 103.33290 | 2399 | 230.27 | 39.07 |
| 28.63202 | 103.29899 | 2672 | 115.43 | 15.31 |
| 28.62058 | 103.31233 | 2680 | 46.40 | 35.20 |
| 28.59662 | 103.27400 | 2644 | 83.03 | 36.32 |
| 28.62825 | 103.34984 | 2159 | 88.53 | 38.34 |
| 28.60175 | 103.35102 | 2382 | 159.78 | 18.18 |
| 28.60360 | 103.36404 | 2365 | 176.08 | 30.68 |
| 28.58460 | 103.38813 | 2203 | 159.27 | 17.78 |
| 28.58411 | 103.39397 | 2218 | 158.36 | 28.79 |
| 28.56861 | 103.34044 | 2029 | 81.77 | 33.08 |
| 28.56220 | 103.34023 | 2198 | 108.88 | 33.59 |
| 28.55685 | 103.34904 | 2087 | 51.34 | 31.92 |
| 28.55119 | 103.33916 | 2480 | 93.69 | 26.74 |
| 28.55066 | 103.33904 | 2486 | 108.05 | 20.78 |
| 28.55360 | 103.33344 | 2440 | 207.23 | 43.20 |
| 28.55697 | 103.31938 | 2733 | 157.70 | 31.34 |
| 28.53852 | 103.35387 | 1982 | 162.24 | 33.08 |
| 28.53408 | 103.32799 | 2559 | 132.51 | 14.79 |
| 28.52207 | 103.30596 | 2239 | 242.75 | 16.75 |
| 28.50484 | 103.32355 | 2184 | 126.59 | 39.45 |
| 28.51846 | 103.26882 | 2521 | 137.19 | 22.88 |
| 28.49438 | 103.26655 | 2408 | 128.47 | 24.12 |
| 28.48383 | 103.31987 | 2620 | 141.71 | 25.49 |
| 28.48152 | 103.34802 | 2504 | 145.28 | 24.07 |
| 28.47214 | 103.26972 | 2563 | 133.21 | 22.68 |
| 28.45682 | 103.26776 | 2625 | 112.93 | 12.89 |
| 28.45843 | 103.27033 | 2904 | 50.53 | 19.65 |
| 28.44855 | 103.29592 | 1750 | 98.46 | 31.24 |
| 28.71610 | 103.30197 | 2283 | 33.92 | 29.54 |
| 28.70957 | 103.28663 | 2800 | 89.70 | 37.46 |
| 28.70800 | 103.25902 | 2869 | 105.32 | 33.42 |
| 28.70619 | 103.24895 | 2752 | 64.93 | 26.01 |
| 28.70524 | 103.24328 | 2088 | 100.10 | 16.73 |
| 28.70706 | 103.24053 | 2814 | 358.03 | 13.24 |
| 28.70691 | 103.24081 | 2797 | 24.78 | 6.62 |
| 28.68652 | 103.21955 | 2737 | 41.68 | 21.62 |
| 28.69902 | 103.22115 | 2702 | 344.06 | 6.73 |
| 28.67721 | 103.21525 | 2705 | 303.23 | 15.70 |
| 28.66466 | 103.20022 | 2642 | 166.70 | 12.90 |
| 28.66673 | 103.14300 | 2640 | 167.74 | 10.80 |
| 28.70003 | 103.12553 | 2772 | 29.93 | 8.78 |
| 28.81848 | 103.17005 | 2618 | 21.25 | 8.90 |
| 28.81516 | 103.15727 | 2840 | 335.32 | 9.37 |
| 28.81325 | 103.16061 | 2674 | 225.00 | 13.85 |
| 28.67946 | 103.08798 | 2830 | 268.60 | 9.44 |
| 28.66865 | 103.07801 | 2585 | 287.93 | 16.16 |
| 28.65346 | 103.08029 | 2437 | 80.48 | 31.49 |
| 28.65581 | 103.04186 | 2870 | 91.71 | 34.11 |
| 28.66187 | 103.01785 | 2673 | 94.71 | 38.28 |
| 28.65971 | 103.02167 | 2478 | 114.52 | 26.93 |
| 28.62851 | 102.95655 | 2783 | 187.37 | 34.80 |
| 28.61702 | 102.93194 | 2652 | 280.31 | 15.21 |
| 28.64287 | 102.88942 | 2790 | 61.70 | 6.83 |
| 28.63380 | 102.90292 | 2960 | 108.07 | 22.05 |
| 28.61148 | 102.90386 | 2911 | 14.91 | 30.25 |
